# Distinction between Neural and Vascular BOLD Oscillations and Intertwined Heart Rate Oscillations at 0.1 Hz in the Resting State and during Movement

**DOI:** 10.1371/journal.pone.0168097

**Published:** 2017-01-04

**Authors:** Gert Pfurtscheller, Andreas Schwerdtfeger, Clemens Brunner, Christoph Aigner, David Fink, Joana Brito, Marciano P. Carmo, Alexandre Andrade

**Affiliations:** 1 Institute of Neural Engineering, Graz University of Technology, Graz, Austria; 2 BioTechMed Graz, Graz, Austria; 3 Institute of Psychology, University of Graz, Graz, Austria; 4 Health Psychology and Applied Diagnostics, University of Wuppertal, Wuppertal, Germany; 5 Institute of Medical Engineering, Graz University of Technology, Graz, Austria; 6 Institute of Biophysics and Biomedical Engineering, Faculty of Sciences of the University of Lisbon, Lisbon, Portugal; University of Cambridge, UNITED KINGDOM

## Abstract

In the resting state, blood oxygen level-dependent (BOLD) oscillations with a frequency of about 0.1 Hz are conspicuous. Whether their origin is neural or vascular is not yet fully understood. Furthermore, it is not clear whether these BOLD oscillations interact with slow oscillations in heart rate (HR). To address these two questions, we estimated phase-locking (PL) values between precentral gyrus (PCG) and insula in 25 scanner-naïve individuals during rest and stimulus-paced finger movements in both hemispheres. PL was quantified in terms of time delay and duration in the frequency band 0.07 to 0.13 Hz. Results revealed both positive and negative time delays. Positive time delays characterize neural BOLD oscillations leading in the PCG, whereas negative time delays represent vascular BOLD oscillations leading in the insula. About 50% of the participants revealed positive time delays distinctive for neural BOLD oscillations, either with short or long unilateral or bilateral phase-locking episodes. An expected preponderance of neural BOLD oscillations was found in the left hemisphere during right-handed movement and unexpectedly in the right hemisphere during rest. Only neural BOLD oscillations were significantly associated with heart rate variability (HRV) in the 0.1-Hz range in the first resting state. It is well known that participating in magnetic resonance imaging (MRI) studies may be frightening and cause anxiety. In this respect it is important to note that the most significant hemispheric asymmetry (p<0.002) with a right-sided dominance of neural BOLD and a left-sided dominance of vascular BOLD oscillations was found in the first resting session in the scanner-naïve individuals. Whether the enhanced left-sided perfusion (dominance of vascular BOLD) or the right-sided dominance of neural BOLD is related to the increased level of anxiety, attention or stress needs further research.

## Introduction

Very slow fluctuations in neural and hemodynamic signals between 0.01 Hz and 0.2 Hz, with a dominant frequency at 0.1 Hz, are characteristic of the resting state [1–4]. The first who reported on slow fluctuation in BOLD oscillations (<0.1 Hz) were Biswal et al. [5]. Recently, 0.1-Hz oscillations were observed presurgically in BOLD signals and intraoperatively in the diameter of specific pial arterioles in the same brain area [6]. These authors suggested that 0.1-Hz oscillations could originate in cerebrovascular tone (vasomotion). Another source for BOLD oscillations might be the 0.1-Hz oscillations in systemic blood pressure (BP) known as Mayer waves [7]. A correlation between Mayer waves in BP and cerebral blood flow velocity (CBFv) in the middle cerebral artery was reported by Diehl et al. [8]. BOLD oscillations driven by Mayer waves in BP are summarized as vascular BOLD oscillations.

Neural BOLD oscillations presume not only the existence of slow intrinsic neural activity oscillations but also a neurovascular coupling. Only a few studies reported on spontaneous neural activity oscillations in electroencephalogram (EEG) and electrocorticogram (ECoG) during rest. Infraslow EEG oscillations in a wide frequency range (0.02–0.2 Hz) with a dominance at 0.1 Hz were documented by Vanhatalo et al. [2] and slow EEG alpha and/or beta power oscillations (0.1 Hz) in sensorimotor areas were reported by Pfurtscheller et al. [9]. Foster and Parvizi [10] described beta, gamma and theta/gamma power modulations with constant spectral peaks distributed around 0.1 Hz (mean 0.1 Hz) at multiple ECoG electrodes placed in the human posteromedial cortex.

With respect to the neurovascular coupling two findings are of interest: First, Bruyns-Haylett et al. [11] reported that a single spontaneous neural spike is followed by a hemodynamic wave peaking about 2–3 s later. Second, Golanow et al. [12] reported on ECoG recording from parietal cortex and laser-Doppler flowmetry (rCBF) over frontal cortex in spinalized rats. They found a high correlation of 0.94 between number of ECoG bursts/min and rCBF waves/min. Of interest is further that the latency between ECoG-onset and 10% increased in rCBF was relatively stable at 2 s, although the intervals between ECoG bursts and rCBF waves varied in a broad range between 7–15 s. Therefore, we assume that neural activity oscillations are associated with BOLD oscillations 2–3 s later. Support for the assumption that neural activity oscillations can drive BOLD oscillations comes from Pfurtscheller et al. [9,13] who observed short-lasting epochs (duration about 100 s) of coupled prefrontal (de)oxyhemoglobin and central EEG alpha/ beta power oscillations in the resting state.

Another point that needs consideration is the mutual interaction between brain and heart enunciated by Claude Bernard already 150 years ago [14]. One classical example of brain-heart coupling is the preparation of voluntary movements characterized by EEG alpha/beta desynchronization (power decrease) and concomitant heart rate (HR) deceleration (RRI increase) observable up to 3 s prior to movement onset [15–17]. Another example is the orienting reflex with a whole body response including EEG alpha desynchronization and short-lasting RRI increase [18,19]. Recently, Thayer and Lane [20] published an extensive review on cortical control of cardiac activity via vagal nerve activation. Furthermore, there is evidence for time-locked fluctuations of slow RRI waves and corresponding EEG alpha/beta power changes during rest, which are also characteristic for movement planning ([13]; see Fig 3 right panel).

These observations lead to the following questions: (i) Is the origin of slow BOLD oscillations with a frequency of around 0.1 Hz vascular or neural? (ii) Is there an interaction between brain and heart activity mediated by slow oscillations? Calculation of the phase-locking (PL) value between BOLD oscillations (0.1 Hz) in brain regions selected due to their arterial vascularization and functional relevance during one-sided hand movement could help answering these questions. Therefore, we selected the precentral gyrus (PCG) and the insula for our investigations. The PCG is part of frontal motor areas and active not only during planning and execution of voluntary movements [15], but also during rest [3,9]. The PCG is bidirectionally connected with the insula via striatum [21] and densely connected to supplementary motor area (SMA; [22]). Both SMA and premotor areas are part of a network with links to middle-posterior insula [23]. The insula plays a putative role in the initiation of movement [23] and is also activated during prefrontal control of cardiac functions [20,24]. Both PCG and insula are supplied by branches of the middle cerebral artery (MCA). From the PL profile the positive (negative) time delay during phase-locked segments can be extracted. A positive time delay (pTD) characterize neural BOLD oscillations preceding in the PCG, whereas a negative delay (nTD) characterize vascular BOLD oscillations preceding in the insula. The nTD stands for time shift of blood flow oscillations at 0.1 Hz with origin in MCA (CBFv = ~ 65 cm/s; [8]) spreading from proximal (insula) to more distal branches (PCG). Here it is expected that the CBFv is slowed down in smaller vessels and cerebral tissue. The pTD defines not the exact time delay between PCG and insula activation, but gives evidence about the spread of slow neural activity oscillations at ~ 0.1 Hz with origin in the sensorimotor network [25] including the PCG and other structures to the tonic alertness network [26] with bilateral insula, anterior cingulum, basal ganglia, thalamus and others. The pTD is characteristic for central commands with the goal to modulate cardiac function and enhance the heart rate variability (HRV).

Our working hypothesis was the following: If intrinsic neural BOLD oscillations (0.1 Hz) exist in PCG and insula, then the driving neural activity oscillations should spread from prefrontal cortex not only to the insula, but should also project to the cardiovascular nuclei in the brain stem. In the case of a more intensive coupling between PCG and insula a strengthened cyclic modulation of HR could be expected as signaled by HRV. This provides evidence that HR [27] can be enhanced not only by Mayer waves in the BP but also through central commands whereby a high HRV represents a type of resource that can be utilized in emotion regulation and therewith also in anxiety processing [20].

In the present study, we used BOLD signals recorded with a high scanning rate in scanner naïve participants to investigate the phase-coupling of 0.1-Hz oscillations in two resting states and one session with cue-paced right hand movements in regular 10-s intervals. One goal of this paper is to use PL computations to differentiate between slow BOLD oscillations of neural or vascular origin in different resting states and during movement. Other goals are to investigate hemispheric asymmetries of phase coupling, the relationship between BOLD oscillations and HRV, and to obtain a first estimate of the velocity of slow vascular BOLD oscillations in small vessels during rest.

## Methods

### Participants

A total of 25 individuals (12 female) between 19–34 years (mean ± SD: 24 ± 3.2 years) took part in the study. All were naïve to the purpose of the study, had no former MRI experience, had normal or corrected-to-normal vision and were without any record of neurological or psychiatric disorders as assessed via self-report. All individuals gave informed written consent to the study protocol, which had been approved by the local Ethics Committee at the University of Graz.

### Experimental protocol

The experimental task started with a first rest fMRI period (R1) lasting approximately 350 s, followed by two movement sessions and a second rest period (R2) lasting also 350 s. In the second movement session (MOV; lasting about 560 s), participants were instructed to press a button with their right hand whenever a visual cue was presented at regular intervals of 10 s. In addition to both rest sessions, only the second movement session was analyzed in the present study. Participants were requested to keep their eyes open, stay awake, and avoid movements during rest.

### fMRI data acquisition and preprocessing

Functional images were acquired on a 3 T scanner (Magneton Skyra, Siemens). A multiband GE- EPI sequence [28,29] was applied with the following parameters: multiband factor 6, voxel size 2x2x2 mm³, TR/TE = 871/34 ms, flip angle 52 degrees, matrix 90x104, 66 contiguous axial slices, FOV = 180x208 mm². 400 volumes (resting state) and 650 volumes (movement session) were acquired during rest. Pre-processing and region of interest (ROI) signal extraction was performed using the DPARSF toolbox [30]. Pre-processing included the removal of the first 10 volumes (to ensure signal stability), slice- timing correction adapted for multiband acquisitions [31], motion correction, normalization to Montreal Neurological Institute (MNI) space, resampling to 2-mm isotropic voxels, spatial smoothing with a 4-mm FWHM Gaussian kernel and linear detrending. Lastly, the BOLD time courses of left and right precentral gyrus and left and right insula were extracted, as defined in the Automated Anatomical Labeling (AAL) atlas [32].

### Physiological data acquisition and processing

An ECG was recorded inside the scanner using the Siemens Physiological ECG Unit. For the positioning of the ECG electrodes on the thorax, standard channels (Siemens Standard, lead 1) were used. The sampling rate was 400 Hz. The FMRIB plug-in for EEGLAB was used for QRS (heart beat) detection [33]. Within this tool, the FASTR algorithm (for removal of gradient-induced artifacts) and the QRS detection algorithm were used in succession, resulting in beat-to-beat interval (RRI) time courses. Those were further interpolated at the same sampling frequency as the BOLD acquisitions (1/871 ms^-1^). The final steps included the calculation of the beat-to-beat interval (RRI) time courses (sample rate 4 Hz) and the HRV spectrum for the band 0 to 0.5 Hz (Kubios HRV version 2.0; [34]). From each spectrum, the percentage of low frequency power in the range 0.07–0.13 Hz was calculated.

### BOLD data processing

Wavelet transform coherence (WTC) was applied to the BOLD time series using the “Cross-wavelet and Wavelet Coherence” toolbox [35]. The Morlet mother wavelet was chosen due to its conceptual simplicity and widespread use. WTC provides a time-frequency map of complex coherence between two signals. While the squared magnitude of the coherence is often used to study the coupling between two signals, here we focus on the phase component, which allows us to compute the phase-locking (PL) value throughout the acquisition interval (except for small sections at the beginning and end, known as the “cone of influence”, where results are known to be unreliable, see [36]). PL is a normalized measure of how much the phase difference between two signals changes in a user-chosen time window, regardless of the actual phase difference value; the reader is referred to [37] for more details. This computation was restricted to frequencies between 0.07 and 0.13 Hz and was performed for every time point with a window size of 4 cycles (corresponding to about 40 s). In order to compute the statistical significance of PL values and thereby test the null hypothesis of independent pairs of oscillatory activity, a surrogate-based method was used [38]. Briefly, 100 surrogates of each time series were created through phase randomization while preserving other relevant properties, notably power spectrum. PL was computed for all surrogate pairs leading to the computation of an empirical statistical distribution and to the p = 0.05 threshold. PL values above the empirically defined, user-chosen threshold were considered significant. The positive (negative) time delay is computed from the phase component of the WTC at each time point and can be averaged across all significant time points in order to provide an average delay (henceforth called *delay*). The percentages of significant time bins, which indicate the total length of the significant phase-locking episodes (henceforth called *%sigbins)*, are also useful indicators of the degree of PL throughout time.

## Results

### Phase-locking (PL) profiles

**Fig 1:**
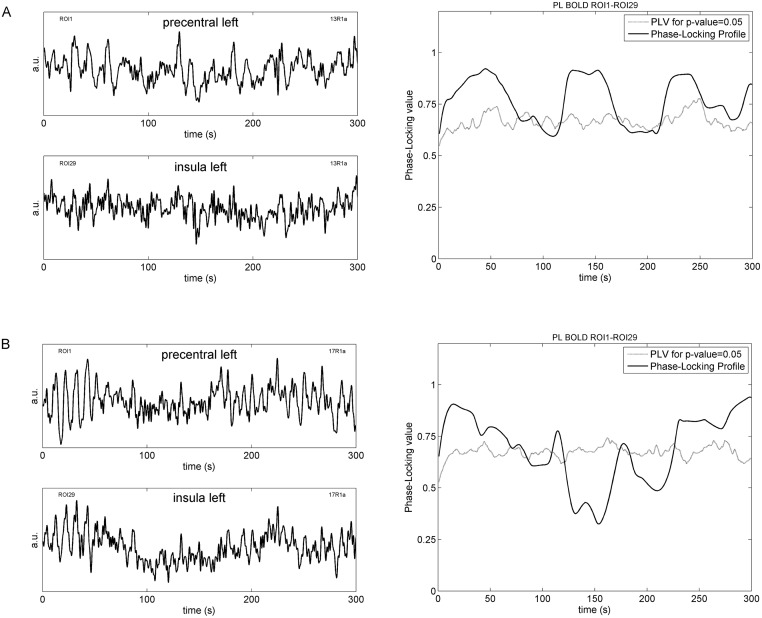
Left side: Examples of BOLD time series (PCG and insula) from two characteristic individuals, one with vascular BOLD (nTD) oscillations (participant 13R1a; A) and one with neural BOLD (pTD) oscillations (participant 17R1a; B). Right side: corresponding PL profiles (i.e., plots of PL across time) with threshold (PLV for p = .05) for an individual with nTD (*delay:* -0.42 s, *%sigbins:* 84%) and an individual with pTD (*delay:* 0.05 s, *%sigbins:* 60%).

Fig 1 displays examples of BOLD time courses from precentral (PCG) and insula (left side) with the corresponding PL profile (right side) during rest R1. The data of two characteristic participants are shown, one with vascular BOLD (upper panel) and one with neural BOLD oscillations (lower panel). A couple of slow waves are clearly visible especially in the subject with neural BOLD signals. Remarkably, the different PL profiles show clear differences between individuals, one of them (participant 13R1a) shows a long coupling time (*%sigbins* = 84%) and fluctuations with periods of about 100 s, the other (participant 17R1a) shows shorter coupling time (*%sigbins =* 60%) and more or less irregular fluctuations.

### Hemispheric asymmetries

**Fig 2:**
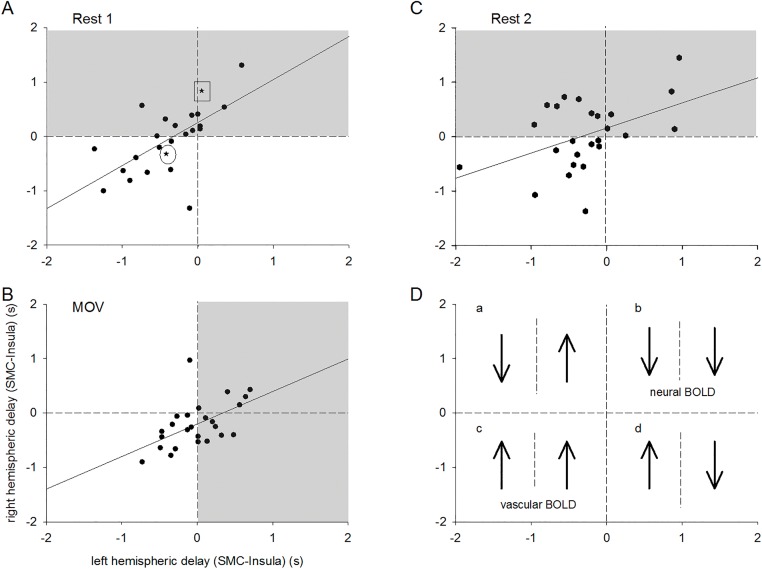
Interhemispheric asymmetry plots of PL delay between SMC and insula for rest 1 (A), movement (B) and rest 2 (C). Gray regions indicate the hemisphere with a majority of neural BOLD signals (pTD). In panel A two representative individuals are marked (rest 1), one (17R1a) with bilateral pTD (indicated by a square) and another (13R1a) with bilateral nTD (indicated by a circle). D: Cartoon illustrates the BOLD direction in the four quadrants (quadrant b: bilateral pTD; quadrant c: bilateral nTD).

From each of the 25 individuals, two pairs of PL parameters (*delay* and *%sigbins*), one from each hemisphere, were extracted for each session (R1, R2 and MOV). The grand averages of the parameters *delay* and *%sigbins* (mean ± SD), separated for each hemisphere and session are summarized in Table 1. In addition, the significance (t-test) of hemispheric differences is indicated. Assumption of normality was met for each variable as verified by Shapiro-Wilk tests, except for duration in R1 for the right hemisphere. Importantly, applying non-parametric statistics (Wilcoxon test) did not result in a different finding. TD values for each period (R1, R2 and MOV) are displayed in the form of interhemispheric scatter plots together with the regression lines in Fig 2. The Spearman inter-hemispheric correlation for all 25 participants was significant during rest 1 (r = .66; p < .001) and during the movement task (r = .57; p < .003). Significance was not reached in rest R2 (r = .33; p = .11).

**Table 1:** Mean (M) and standard deviation (SD) of PL *delay* and *%sigbins* in each hemisphere (25 participants). Indicated are difference (D), t-value, degrees of freedom (df) and significance (p) of hemispheric differences. Data from rest 1 (R1), rest 2 (R2) and movement (MOV) sessions.

		Left hemisphere		Right hemisphere					
		*M*	*SD*	*M*	*SD*	*D*	*t*	*df*	*p*
R1	delay (s)	-.38	.47	-.05	.59	-0.33	-3.54	24	.002
R1	%sigbins	52.2	25.2	37.9	22.5	14.3	3.31	24	.003
R2	delay (s)	-.28	.62	-.03	.63	-0.25	-2.35	24	.03
R2	%sigbins	45.8	22.8	38.6	24.0	7.2	1.91	24	.07
MOV	delay (s)	-.0008	.38	-.20	.42	0.20	2.62	24	.02
MOV	%sigbins	52.6	23.9	60.6	22.9	-8.0	-1.86	24	.08

Significant hemispheric differences were found for *delay* during R1, R2 and movement (MOV). In addition, *%sigbins* was larger in the left hemisphere during rest (R1, R2) and in the right hemisphere during MOV. More detailed information about the hemispheric asymmetry can be retrieved from Fig 2. There were more pTD in the right than in the left hemisphere during both R1 and R2, and more pTD in the left hemisphere than in the right hemisphere during movement (grey regions in Fig 2, Table 2). Hence, irrespective of hemispheric differences and task periods pTD signaling neural BOLD oscillations could be found in a relatively large proportion of participants (approximately 50%).

**Table 2:** Spearman correlation coefficient (r) and significance (p) between *%sigbins* and HRV in the 0.1 Hz band for rest (R1, R2) and movement (MOV). The respective numbers of participants (n) with positive (negative) time delays in the right (left) hemisphere are indicated. For example: in R1 were 13 pTD in the right and 7 pTD in the left hemisphere. The subjects indicated by bold numbers are used for correlation calculations.

	Positive time delay				Negative time delay			
Task	left	right	*r*	*p*	left	right	*r*	*p*
R1	7	**13**	.67	.01	**18**	12	.47	.15
R2	6	**13**	.59	.03	**19**	12	.40	.09
MOV	**13**	6	.51	.07	12	**19**	.33	.17

### Correlation between BOLD (%sigbins) and HRV

**Fig 3:**
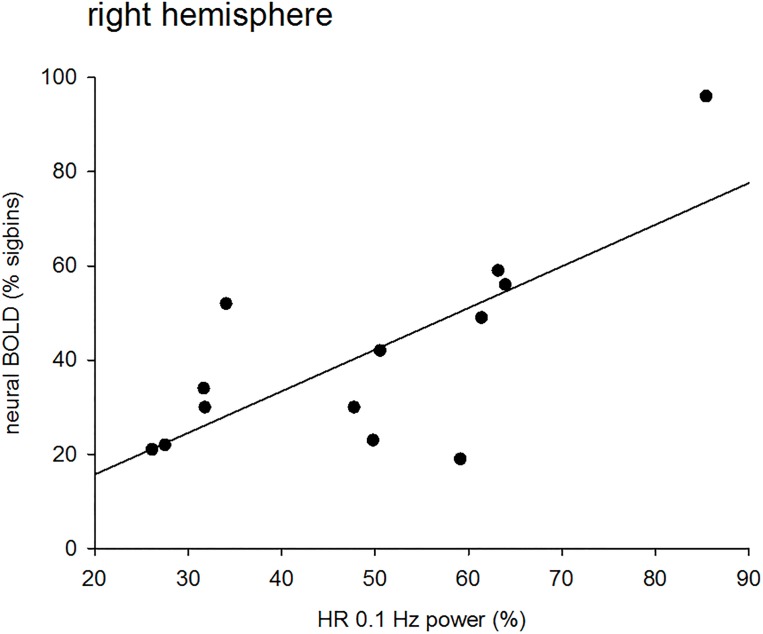
Significant positive correlation (p<0.01) between neural BOLD (*%sigbins*) and HRV (0.1-Hz power) in the right hemisphere of subjects with pTD in R1.

To evaluate our working hypothesis, it was necessary to separate individuals with pTD (neural BOLD) and nTD (vascular BOLD) and calculate the correlation coefficient between *%sigbins* and percentage spectral power of the HRV in the band 0.07–0.13 Hz for rest (R1 and R2) and movement (MOV). Correlations are summarized in Table 2. The correlation between neural BOLD and HRV was significant for the right hemisphere during R1 (see example in Fig 3) and R2, and approached significance for the left hemisphere during MOV. The numbers indicate the quantity of participants with hemisphere-specific nTD or pTD. No correlation was found between vascular BOLD and HRV in the left hemisphere during rest and in the right hemisphere during movement.

## Discussion

The aim of this study was to differentiate between slow BOLD oscillations of primary neural or vascular origin during rest and movement by using PL computations. We found evidence for a separation between neural and vascular BOLD responses based on correlations with HRV.

### Hemispheric asymmetry

During the movement task a significant hemispheric asymmetry for phase-locking was found with a larger number of pTD in the left then right hemisphere (Table 2; left: 13 pTD, right: 6 pTD). These findings are expected, because right-hand button press is a well-defined motor task accompanied by preponderance of EEG [15,39] and BOLD responses in the left hemisphere (3). In this respect, the dominance of the neural BOLD oscillations (pTD) in the left hemisphere and the corresponding correlation with HRV (Table 2) is plausible. Although the movement task was relatively simple (stimulus-paced button press in regular 10 s intervals), the ongoing hemodynamic responses can display a considerable variability depending on whether the movement-evoked oscillations at 0.1 Hz were entrained or not to the intrinsic oscillations at subject-specific frequency around 0.1 Hz. This variability caused by the partial superposition of intrinsic and evoked BOLD oscillations may explain the non-significant (r = .51, p = .07) correlation between neural BOLD and HRV.

Remarkably, a hemispheric asymmetry was also observed in the resting states (Table 1), which was most pronounced in R1. Notably, contrary to the movement session there was a dominance of pTD in the right hemisphere (n = 13) and nTD in the left hemisphere (n = 18; Table 2). This asymmetry in the first resting session could be explained by the fact that all participants were scanner-naïve. It is well known that participating in MRI studies could be frightening and cause anxiety [40]. The preponderance of neural BOLD oscillations in the right side and the predominant vascular BOLD oscillations in the left side warrants further research. Whether the former is related to the right hemispheric innervation of the sinoatrial node of the heart and emotion processing [20] and the latter to an enhanced left hemispheric perfusion during anxiety and stress, needs further investigation. Hemispheric asymmetries have been reported in relation to anxiety [41] and different types of emotional processing [42,43].

### Neural BOLD oscillations and HRV

The correlation between right-sided episodes of *%sigbins* (pTD) and HRV (0.1-Hz spectral power) was significant during R1 only (p < .01; Table 2) and approached significance for the left hemisphere in the movement task. Specifically, participants with pTD and longer phase-locking episodes (larger *%sigbins*) between PCG and insula displayed also a larger HRV and vice versa. Such a high HRV especially in the first resting state (R1) in scanner- naïve subjects with a high anxiety level is important for a successful regulation of unpleasant emotions [20]. The existence of neural BOLD oscillations in premotor areas is also supported by the recently reported finding of a significant temporary coupling of prefrontal oxyhemoglobin and central beta (mu) power oscillations at ~ 0.1 Hz during rest [9,13]. It should be noted though, that replication studies are certainly warranted in order to evaluate the robustness of this finding. The statistical power was rather low and results could have been influenced by a single individual showing high values on both measures (see, Fig 3).

### Vascular BOLD signals and perfusion

Vascular BOLD oscillations at 0.1 Hz are generated by a complex interplay of slow cerebral blood volume and CBFv oscillations [1]. Diehl et al. [8] reported a CBFv of ~ 65 cm/s measured in the MCA by transcranial Doppler sonography. To reveal an estimate of the velocity of vascular BOLD waves in branches of the MCA and surrounding cerebral tissue the mean nTD with reliable phase coupling of %sigbins> = 10% was calculated for the left hemisphere in R1. In contrast to the mean TD across all participants of -0.38 s (see Table 1, R1) the mean nTD using only participants with %sigbins > = 10% was ~ -0.6 s. Assuming an estimated distance of 8 cm between the branches of MCA in insula and PCG this corresponds to a velocity of vascular BOLD oscillations of ~ 13 cm/s. Common for both velocities (~65 cm/s in MCA and ~13 cm/s in small vessels) is that they are driven by the Mayer waves in the BP.

### Limitations of the study and future perspectives

In studies with slow BOLD oscillations artefacts have to be considered. Structured noise, including especially cardiac and respiratory related artefacts, is one of the main contributors of BOLD signal correlations across different brain areas [44]. Due to long TR used in usual scanning protocols (more than 1s), the effects of respiratory and cardiac artefacts are aliased into lower frequencies. Our Nyquist frequency is 0.57 Hz, hence there is no risk of aliasing of respiratory effects (~0.3 Hz) into our band of interest (0.07–0.13 Hz).

Furthermore, we think that no plausible scenario would account for the hemispheric asymmetries that we have found, if the BOLD signals were dominated by a common cardiac driver. Besides, removing the cardiac effects in this case (using e.g. Retroicor [45]) might be counterproductive, because it could remove the HR variability effects that we rely upon for some of our conclusions.

A limitation of the study is that respiration was not considered. For further similar studies of BOLD-RRI phase coupling the analyzing of breathing signals is recommended, because especially low frequency components of BOLD oscillations could be confounded by the breathing cycle [46].

Further research should focus on issues such as the following, already mentioned: hemispheric asymmetry, low level of statistical power of correlations with HRV oscillations and estimation of intracerebral CBF velocity also in the supply area of the internal cerebral artery. It is important to study lower (< = 0.1 Hz) and upper (f>0.1 Hz) frequency bands, because all available evidence indicates that BOLD oscillations of neural origin are essentially absent above 0.1 Hz [44], to analyze more resting state with quantified anxiety states and to compute the phase shift between BOLD and RRI oscillation in the 0.1-Hz band. Concerning the last point, recently published work [47] strongly suggests that vascular BOLD and neural BOLD oscillations can be differentiated by the help of RRI oscillations at 0.1 Hz.

### Conclusion

The PL method is a powerful tool not only to study phase synchrony in neural oscillations of various frequency components [37,38], but could also enable a distinction between BOLD oscillations of neural or vascular origin.

Right-hand movements in regular intervals of 10 s are accompanied by EEG alpha/beta desynchronization and evoked BOLD responses with a preponderance in the left sensorimotor area. This means that neural activation starting in the prefrontal cortex and spreading to more proximal areas (e.g., insula and cardiovascular nuclei) is characteristic for neural BOLD oscillations and pTD, respectively. This holds also for the resting state with a remarkable right-sided dominance of spontaneous neural BOLD oscillations that were accompanied by elevated HRV. The link between spontaneous neural BOLD and neural activity oscillations, respectively, and HRV was demonstrated the first time in this paper, but needs further research, for example by computing PL profiles for slow BOLD and RRI oscillations measured simultaneously. In contrast to the neural BOLD oscillations driven by neural activity changes, vascular BOLD oscillations are driven by Mayer waves in the BP. Both types of BOLD oscillations have a dominant frequency at approximately 0.1 Hz and can be superimposed, whereupon only one component (neural or vascular) will be dominant. This dominance seems to vary from resting state to resting state and may depend on the level of awareness, attention and other factors.

Further research is needed to investigate the hemispheric asymmetry of neural and vascular BOLD oscillations and innervation of the heart, to study the entrainment effect of movements in regular intervals to ongoing BOLD signals, and to analyze the stability of phase-locking.
